# Unique endoscopic features of primary biliary diffuse large B‐cell lymphoma: A case report with literature review (with video)

**DOI:** 10.1002/deo2.414

**Published:** 2024-07-28

**Authors:** Tomoya Nakamura, Yoshiharu Masaki, Naohiro Kameyama, Yujiro Kawakami, Keisuke Ishigami, Yumemi Takada, Shuji Satoh, Taro Sugawara, Shintaro Sugita, Hiroshi Nakase

**Affiliations:** ^1^ Department of Gastroenterology and Hepatology Sapporo Medical University School of Medicine Hokkaido Japan; ^2^ Department of Gastroenterology and Hepatology Obihiro Kyokai Hospital Hokkaido Japan; ^3^ Department of Surgical Pathology Sapporo Medical University School of Medicine Hokkaido Japan

**Keywords:** biliary malignant lymphoma, diffuse large B‐cell lymphoma, malignant biliary obstruction, endoscopic ultrasonography, intraductal ultrasonography

## Abstract

A 67‐year‐old man visited our hospital complaining of dark‐colored urine and upper abdominal pain. Magnetic resonance cholangiopancreatography showed stricture of the distal bile duct, and contrast‐enhanced computed tomography showed irregular thickening of the distal bile duct wall. However, no enlarged lymph nodes, pancreatic tumors, or other neoplastic lesions were apparent around the bile duct. Endoscopic ultrasonography and intraductal ultrasonography showed irregular thickening of the inner hypoechoic layer without the disappearance of the innermost thin hyperechoic layer. On the basis of these findings, we considered that the bile duct lesion was of non‐epithelial origin. Thus, we repeatedly performed bile duct biopsies from the same site under fluoroscopy to obtain a sample of the submucosal tissue. The pathological diagnosis was diffuse large B‐cell lymphoma, and the patient received systemic chemotherapy (rituximab, cyclophosphamide, doxorubicin, vincristine, and prednisone). After six courses of rituximab, cyclophosphamide, doxorubicin, vincristine, and prednisone, positron emission tomography‐computed tomography showed the disappearance of 18‐fluorodeoxyglucose uptake in the bile duct and endoscopic retrograde cholangiography showed improvement of the bile duct stricture. Endoscopic findings and repeated biopsies were useful in making the diagnosis of primary biliary diffuse large B‐cell lymphoma.

## INTRODUCTION

Primary biliary malignant lymphoma (PBML) that causes biliary obstruction is extremely rare.^1^ The endoscopic findings of PBML are still unclear; therefore, preoperative diagnosis is difficult, and most patients are diagnosed using postoperative pathology.^2^ However, malignant lymphoma can be cured with non‐surgical treatments, such as chemotherapy, radiotherapy, and chemoradiotherapy. Thus, it is important to diagnose PBML using non‐surgical methods, such as endoscopic biopsy.

Herein, we describe a patient with primary biliary diffuse large B‐cell lymphoma (DLBCL), who showed unique findings on endoscopic ultrasonography (EUS) and intraductal ultrasonography (IDUS).

### Case report

**FIGURE 1:**
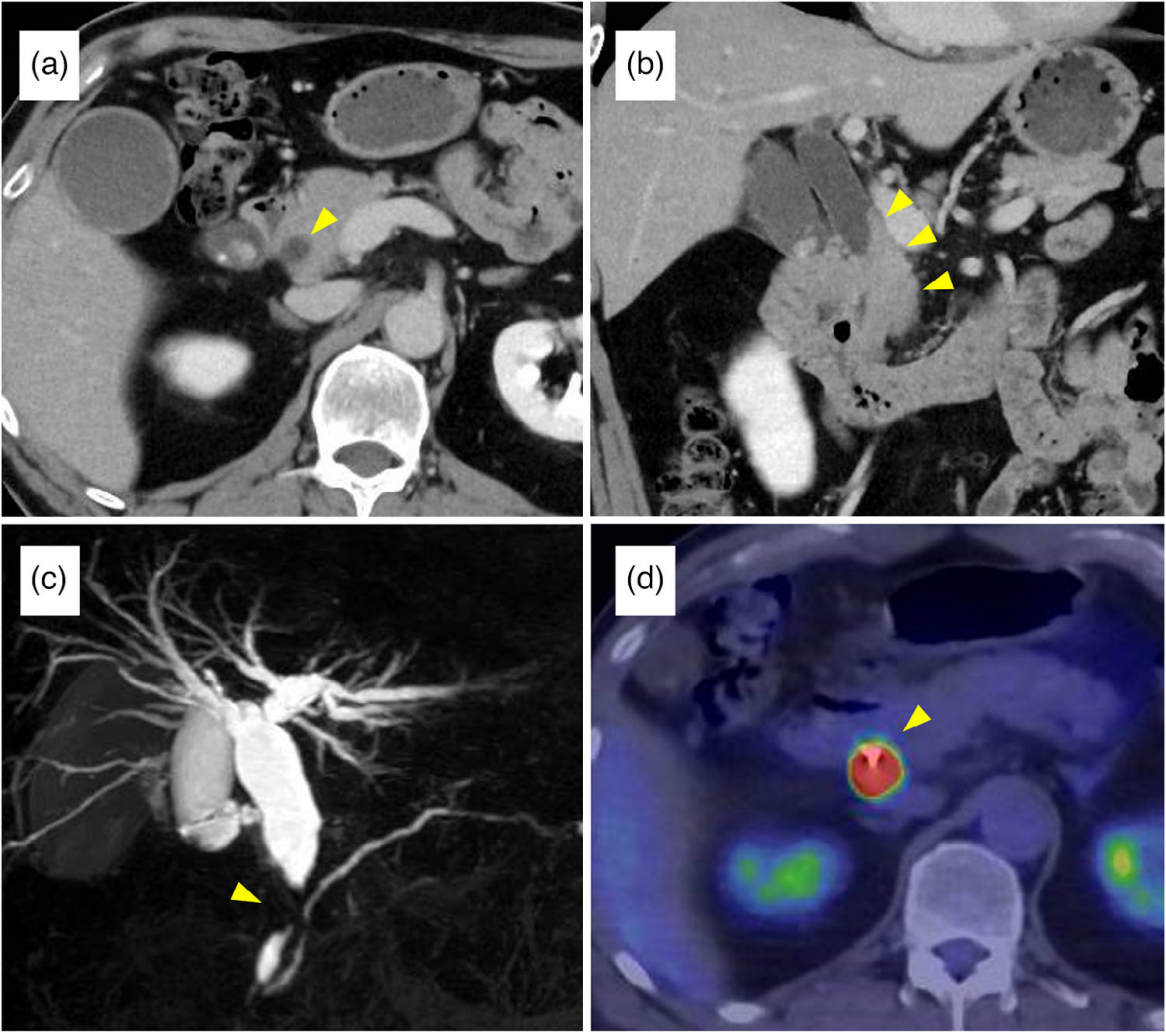
Radiological findings of the bile duct lesion: (a, b) Axial and coronal contrast‐enhanced computed tomography images showing irregular thickening of the distal bile duct wall. (c) Magnetic resonance cholangiopancreatography reveals stricture of the distal bile duct with dilatation of the proximal bile duct. (d) Positron emission‐computed tomography reveals abnormal 18‐fluorodeoxyglucose uptake at the site of the bile duct stricture. The maximum standardized uptake value is 32.29.

**FIGURE 2:**
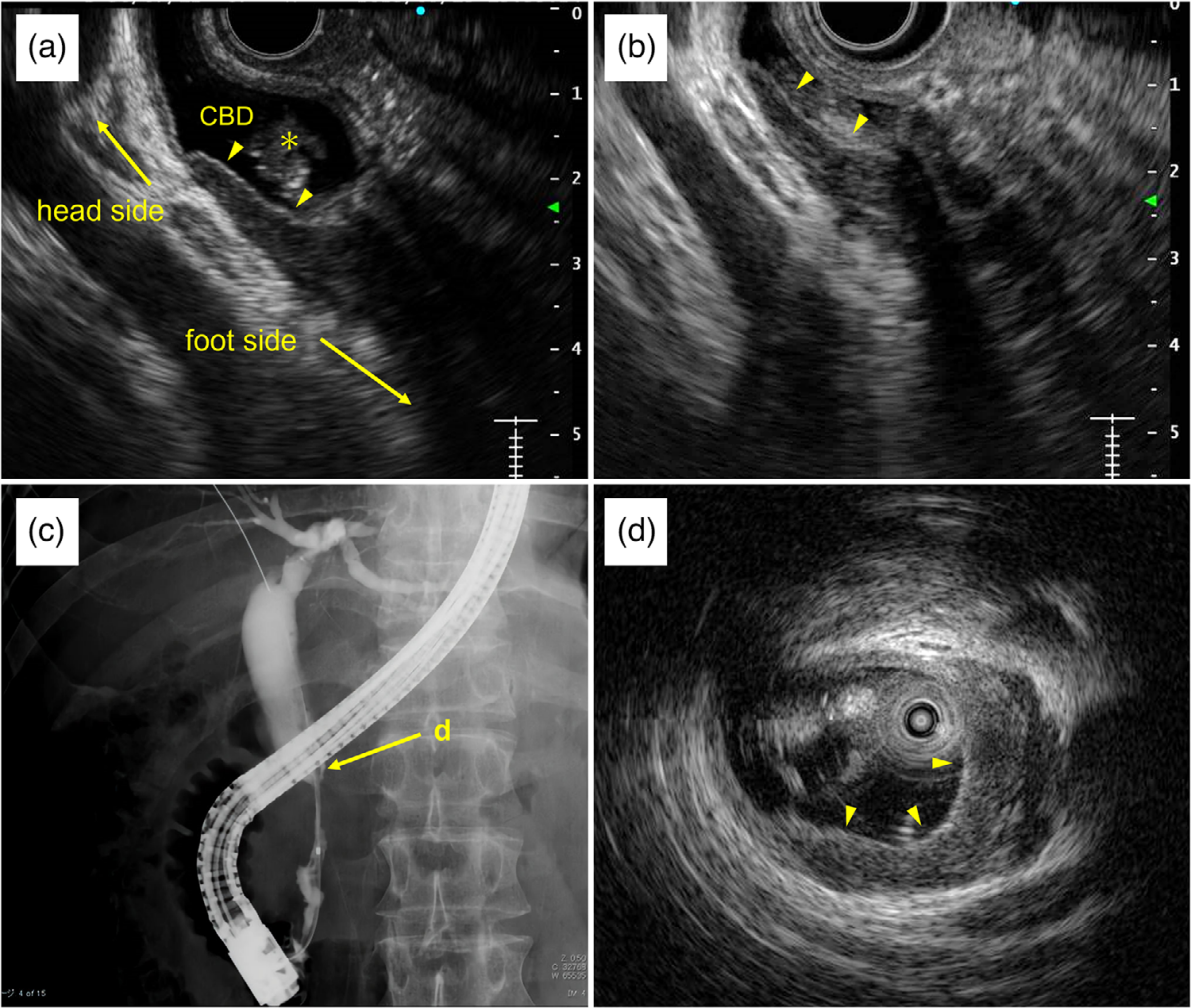
Findings of endoscopic ultrasonography (EUS), endoscopic retrograde cholangiography (ERC), and intraductal ultrasonography (IDUS): (a, b) EUS reveals irregular thickening of the inner hypoechoic layer without the disappearance of the innermost thin hyperechoic layer (arrow). A hyperechoic mass indicating a bile duct stone is also detected (*). (c) ERC shows the distal bile duct stricture with a dilated proximal bile duct. (d) IDUS of the bile duct stricture site shows irregular thickening of the inner hypoechoic layer without the disappearance of the innermost thin hyperechoic layer, as seen on endoscopic ultrasonography.

**FIGURE 3:**
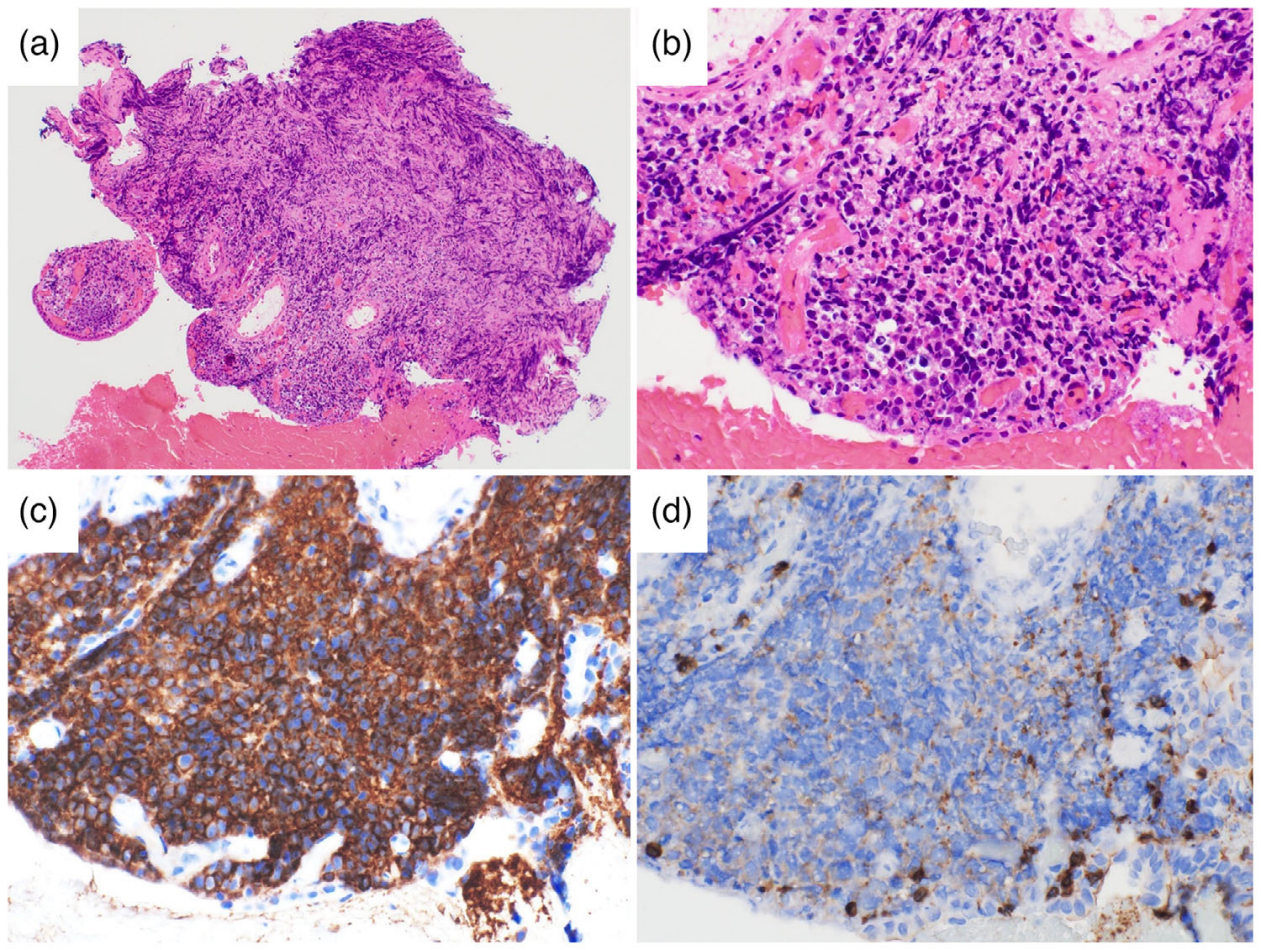
Pathological findings of bile duct biopsy specimens: (a, b) Hematoxylin and eosin staining shows diffuse proliferation of large atypical lymphocytes with irregular nuclear shapes (a: ×100, b: ×400). (c, d) Immunohistochemical staining shows that the lymphocytes were positive for CD20 and negative for CD3 (c: CD20 ×400, d: CD3 ×400).

A 67‐year‐old man visited our hospital complaining of dark‐colored urine and upper abdominal pain. He had a history of asthma and hypertension, but no history of abdominal surgery. The physical examination did not reveal conjunctival icterus, superficial lymphadenopathy, abdominal tenderness, or hepatosplenomegaly. Laboratory tests showed normal levels of total bilirubin (0.9 mg/dL; reference range, 0.4–1.5 mg/dL) and lactate dehydrogenase (219 U/L; reference range, 124–222 U/L) but elevated levels of transaminases (aspartate aminotransferase level, 43 U/L [reference range, 13–30 U/L]; alanine aminotransferase level, 92 U/L [reference range, 10–42 U/L]), alkaline phosphatase level (191 U/L; reference range, 38–113 U/L) and gamma‐glutamyl transferase (764 U/L; reference range, 13–64 U/L). Serum levels of carcinoembryonic antigen, carbohydrate antigen 19‐9, and soluble interleukin‐2 receptor were negative. Additionally, the serum immunoglobulin‐G4 level was normal (47.6 mg/dL; reference range, 11–121 mg/dL). Magnetic resonance cholangiopancreatography showed a stricture in the distal bile duct with dilatation of the proximal bile duct. Contrast‐enhanced computed tomography (CT) revealed irregular thickening of the distal bile duct wall; however, no enlarged lymph nodes, pancreatic tumors, or other neoplastic lesions around the bile duct were apparent (Figure 1a–c, S1). Positron emission tomography‐CT showed abnormal 18‐fluorodeoxyglucose uptake at the site of the bile duct stricture, with a maximum standardized uptake value of 32.29 (Figure 1d). EUS revealed irregular thickening of the inner hypoechoic layer of the bile duct without the disappearance of the innermost thin hyperechoic layer (Figure 2a,b). Endoscopic retrograde cholangiography (ERC) showed a distal bile duct stricture with a dilated proximal bile duct (Figure 2c), and subsequently, IDUS revealed irregular thickening of the inner hypoechoic layer without disappearance of the innermost thin hyperechoic layer, as shown by EUS (Figure 3d). On the basis of the EUS and IDUS findings, we considered that the bile duct lesion was of non‐epithelial origin and repeatedly performed 10 times bile duct biopsies, using the biopsy forceps (Radial Jaw ^TM^; Boston Scientific), aiming the same level under fluoroscopy to obtain a sample of the submucosal tissue (video S3). Pathological findings (Figure 3a–d) revealed a diffuse proliferation of large atypical lymphocytes with irregular nuclear shapes. Immunohistochemical staining showed that the lymphocytes were positive for B‐cell markers (CD20 and CD79a) and negative for T‐cell markers (CD3 and CD5). Therefore, we diagnosed the patient with DLBCL (Lugano classification stage I, and the revised International Prognostic Index score of 1).

The patient received endoscopic biliary drainage (8.5Fr × 7cm plastic stent [Flexima; Boston Scientific]) followed by systemic chemotherapy (rituximab, cyclophosphamide, doxorubicin, vincristine, and prednisone [R‐CHOP]). After six courses of R‐CHOP, positron emission tomography‐CT showed the disappearance of 18‐fluorodeoxyglucose uptake in the bile duct, and contrast‐enhanced CT and endoscopic retrograde cholangiography showed improvement in the bile duct stricture, (Figure S2). Follow‐up has been performed with contrast‐enhanced CT every 3–6 months.

## DISCUSSION

Herein, we describe a rare case of primary biliary DLBCL, diagnosed pathologically after a detailed endoscopic examination. The EUS and IDUS findings in this case accurately reflect the characteristics of PBML as a non‐epithelial lesion. To our knowledge, this is the first report showing the details of EUS and IDUS findings in a patient with primary biliary DLBCL.

The gastrointestinal tract is the most common primary site of extranodal lymphoma,^3^ but malignant lymphoma arising from the extrahepatic bile duct is extremely rare. In addition to the 32 cases summarized by Kato et al.^2^ from 1982 to 2015, 11 cases were reported by 2023, and a total of 43 cases of PBML were reported. Patient characteristics are presented in Table 1. Twenty‐seven patients were male and 16 were female, with a median age of 59 years (range 4–86 years). The most frequent type of lymphoma was DLBCL (34.9 %). Notably, 88.4 % (38 cases) of all patients were diagnosed by postoperative pathology, and only four were diagnosed by endoscopic bile duct biopsy.^4^, ^5^, ^6^, ^7^

**TABLE 1:** Characteristics of previously reported patients with primary biliary malignant lymphoma.

	*n* = 43
Age, median (range)	59 (4–86)
Male:female sex	27:16
Type of lymphoma	
DLBCL	15 (34.9%)
MALT lymphoma	8 (18.6%)
Follicular lymphoma	5 (11.6%)
Other/unknown	15 (34.9%)
Diagnosis	
Postoperative pathology	38 (88.4%)
Endoscopic biopsy^†^	4 (9.3%)
Percutaneous liver biopsy^‡^	1 (2.3%)

^†^ Biopsies were performed under fluoroscopy in 3 patients, and under cholangioscopy in 1 patient.

^‡^ Liver biopsy of the site of liver infiltration was performed.

Abbreviations: DLBCL, diffuse large B‐cell lymphoma; MALT, mucosa‐associated lymphoid tissue

Malignant gastrointestinal lymphoma, which is classified as a non‐epithelial lesion, arises from the submucosal tissue and shows a submucosal tumor‐like appearance, especially in the early phase.^8^ It is considered that PBML shows similar features as gastrointestinal lymphoma. The characteristic of cholangiography in PBML is smooth bile duct stricture, which reflects a submucosal tumor‐like feature, but it is difficult to distinguish it from other diseases such as cholangiocarcinoma by findings of cholangiography alone. Therefore, it is important to combine with the other modalities such as EUS and IDUS. In the present case, EUS and IDUS findings showed irregular thickening of the inner hypoechoic layer of the bile duct without the disappearance of the innermost thin hyperechoic layer. On ultrasonography findings, the innermost hyperechoic layer reflects the mucosa of the bile duct, and the second hypoechoic layer reflects the fibromuscular layer or deeper.^9^ Thus, we consider the EUS and IDUS findings in this case to accurately reflect the characteristics of PBML as a non‐epithelial lesion. Actually, the previous report describing pathological findings of PBML^2^ shows the growth of tumor cells under normal biliary epithelium. The thin hyperechoic layer in the findings of EUS and IDUS represents the normal biliary epithelium, while the irregular thickened hypoechoic layer represents a subepithelial tumor.

Differential diagnoses for PBML include cholangiocarcinoma, immunoglobulin G4‐related sclerosing cholangitis (IgG4‐SC), and primary sclerosing cholangitis (PSC). Cholangiocarcinoma arises from the mucosal epithelium and invades horizontally and vertically; therefore, EUS and IDUS findings show irregular bile duct wall thickening with loss of the three‐layered structure (high‐low‐high echoic layers).^5^ IgG4‐SC manifests with the thickening of the second hypoechoic layer without the disappearance of the three‐layered structure, similar to PBML.^10^ However, the thickening in IgG4‐SC is uniform and has an entire circumference, whereas that in PBML is irregular and unilateral. PSC is characterized by an irregular bile duct lumen and diverticular‐like protrusion due to severe inflammation of the bile duct mucosa, whereas PBML features a smooth bile duct lumen.^10^ When EUS and IDUS findings suggest a non‐epithelial tumor, for example, PBML, repeated biopsies from the same site, the so‐called boring biopsy, may be useful for obtaining submucosal tissue for diagnosis. In this case, we simply performed biopsies aiming at the same level of bile duct under fluoroscopy using a large cup of biopsy forceps to make an effort to obtain submucosal bile duct tissue, therefore it may not have been possible to perform boring biopsies accurately. We consider that it is better to perform the biopsies under cholangioscopy, if possible. As for the number of biopsies, in the previously reported case,^7^ the first and second bile duct biopsies (each four times) did not lead to diagnosis, and the third biopsies (six times) were conclusive. Although it is difficult to determine the adequate number of biopsies, we consider that at least 4–5 times biopsies with large cup biopsy forceps may be necessary.

Among the previous four patients diagnosed by endoscopic biopsy, one patient received radiotherapy and three patients received chemotherapy. Malignant lymphoma can be cured by non‐surgical treatments, such as chemotherapy, radiotherapy, and chemoradiotherapy; in contrast, cholangiocarcinoma can be cured only by surgery. Therefore, the diagnosis of PBML based on endoscopic and biopsy findings is important to avoid unnecessary surgical treatment.

In conclusion, we described a case of primary biliary DLBCL in which endoscopic findings and repeated biopsies were useful for making the diagnosis. Further investigation is needed to establish the endoscopic findings of rare types of biliary lesions such as malignant lymphoma.

## CONFLICT OF INTEREST STATEMENT

None.

## ETHICS STATEMENT

Approval of the research protocol by an Institutional Reviewer Board is not applicable.

## PATIENT CONSENT STATEMENT

Informed consent was obtained from the patient for publication of this case report.

## Supporting information

Figure S1: Each enhance level image of contrast‐enhanced CT

Figure S2: Findings of ERC, contrast‐enhanced CT, and positron emission‐CT before and after treatment

Video: Video of ERC with repeated bile duct biopsies
