# Supplementary material for: Unique endoscopic features of primary biliary diffuse large B‐cell lymphoma: A case report with literature review (with video)
Source: DEN Open. 2024 Jul 28;5(1):e414. doi: 10.1002/deo2.414 (PMC11284119; doi:10.1002/deo2.414)
Supplement: Supplementary file 1 — Figure S1: Each enhance level image of contrast‐enhanced CT [file DEO2-5-e414-s002.docx]

Supporting information


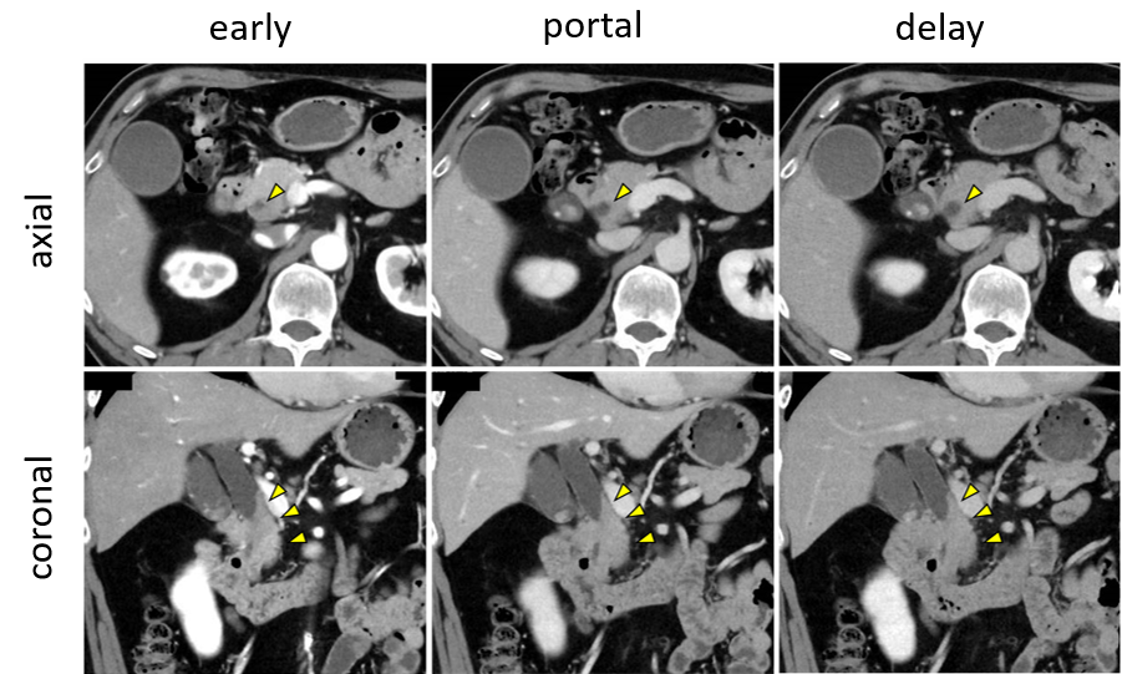


Figure S1: Each enhance level image of contrast-enhanced CT

Contrast-enhanced CT shows irregular thickening of the distal bile duct wall with gradual enhancing.
