# Supplementary material for: Unique endoscopic features of primary biliary diffuse large B‐cell lymphoma: A case report with literature review (with video)
Source: DEN Open. 2024 Jul 28;5(1):e414. doi: 10.1002/deo2.414 (PMC11284119; doi:10.1002/deo2.414)
Supplement: Supplementary file 2 — Figure S2: Findings of ERC, contrast‐enhanced CT, and positron emission‐CT before and after treatment [file DEO2-5-e414-s003.docx]

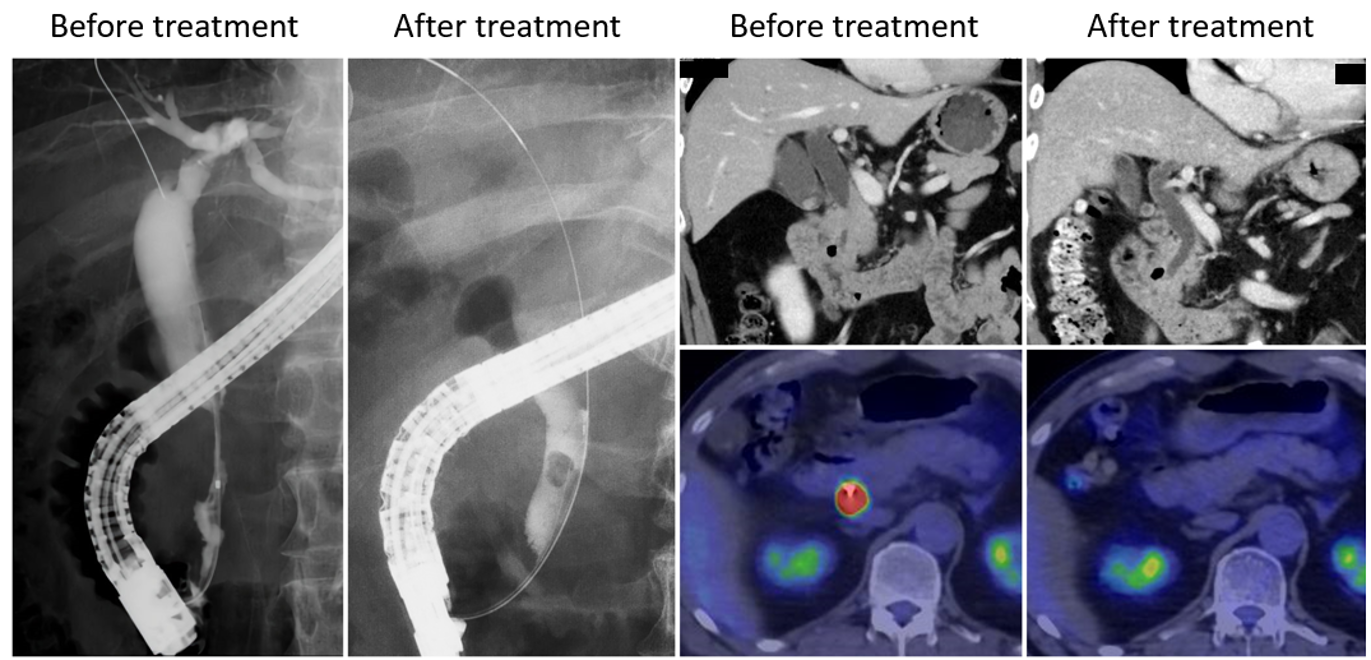


Figure S2: Findings of ERC, contrast-enhanced CT, and positron emission-CT before and after treatment.

ERC performed after chemotherapy shows the improvement of distal bile duct stricture.

Contrast enhanced-CT and positron emission -CT images taken after chemotherapy also show improvement of bile duct stricture and disappearance of FDG uptake in the bile duct.

Video: Video of ERC with repeated bile duct biopsies

We performed repeated biopsies (10 times) aiming the same level of bile duct under fluoroscopy using a large cup biopsy forceps (Radial Jaw^TM^; Boston Scientific).
